# Fatty acid composition of subcutaneous adipose tissue and gastric mucosa: is there a relation with gastric ulceration?

**DOI:** 10.1186/1471-230X-9-9

**Published:** 2009-01-23

**Authors:** Vasileios A Pagkalos, Joanna Moschandreas, Michael Kiriakakis, Maria Roussomoustakaki, Anthony Kafatos, Elias Kouroumalis

**Affiliations:** 1 Department of Gastroenterology, University Hospital of Heraklion, Heraklion, Crete, Greece; 2 Department of Social Medicine, Preventive Medicine and Nutrition, Medical School, University of Crete, Heraklion, Crete, Greece

## Abstract

**Background:**

Both in vitro and epidemiological studies indicate that dietary polyunsaturated fatty acids may play a protective role against peptic ulcer in humans. Adipose tissue fatty acid composition is thought to reflect dietary fatty acid intake. The aim of the present study is to investigate adipose and gastric mucosa fatty acid levels in relation to gastric ulceration status.

**Methods:**

Fifty two adult outpatients undergoing upper gastrointestinal tract endoscopy participated in the study. Adipose tissue samples were taken from the abdomen and buttock during the endoscopy procedure and samples from gastric tissue were taken from a subsample of 30 subjects. The presence of *Helicobacter pylori *was determined using the CLO test. Capillary gas chromatography was used for the extraction of 36 and 42 adipose tissue and gastric mucosa lipids respectively.

**Results:**

The monounsaturated fatty acids (MUFAs) C18:1*n*-12c, C16:1*n*-5, C16:4*n*-1 and the polyunsaturated fatty acids (PUFAs) C16:3*n*-4, C20:3*n*-3, C20:4*n*-6, C21:5*n*-3 and C18:2*n*-9c,12t of the gastric mucosa were present in higher proportions in ulcer negative patients. These unsaturated fatty acids, however, each contributed less than 1% on average to total fatty acid content. In addition, higher average levels of eicosapentaenoic acid (EPA) C20:5*n*-3 and docosahexaenoic acid (DHA) C22:6*n*-3 were detected in abdominal and buttock samples in CLO negative controls, compared to CLO positive controls. Adipose tissue and gastric mucosa *n*-6 and *trans *fatty acid levels were positively linearly correlated (r = 0.37 and 0.41 for *n*-6 and *trans *fatty acids respectively).

**Conclusion:**

Certain minor MUFAs and PUFAs of the gastric mucosa appear to be present in higher proportions in ulcer negative patients. Overall, the findings provide only weak evidence of an association between the gastric mucosal fatty acids and the presence of gastric ulceration. The higher average levels of EPA and DHA in abdominal and buttock adipose tissue in CLO negative controls could be an indicator that dietary FAs inhibit *Helicobacter pylori *growth. Larger studies are necessary to provide evidence of a biologically relevant effect.

## Background

For more than a century, peptic ulcer disease has been a major cause of morbidity and mortality. The pathophysiology of peptic ulcer disease has centered on an imbalance between aggressive and protective factors in the stomach [1].

Twenty five years have elapsed since Marshall and Warren's discovery of the link between *Helicobacter pylori *(*H. pylori*) infection and peptic ulcer disease [2]. The clinical outcome of *H. pylori *infection is most likely the result of complex interactions between bacterial, environmental, and host-related factors [3,4]. The prevalence of *H. pylori *infection varies, has been decreasing in the last few decades in most developed countries [4]. Epidemiological evidence suggests that the declining prevalence of peptic ulcer disease may be partially attributable to increased consumption of polyunsaturated fatty acids (PUFAs), a hypothesis supported by in vitro evidence of toxicity of such substances to *H. pylori *[5].

It has therefore been suggested that dietary fat plays a protective role against peptic ulcer disease. The fatty acids (FAs) present in adipose tissue include certain fatty acids that cannot be endogenously synthesized and are, consequently, considered valid biomarkers of the dietary intake of these FAs [6]. Since adipose tissue has a slow turnover, it is an attractive choice for the study of long-term dietary fatty acid intake [6]. Fatty acids that cannot be synthesized endogenously from carbohydrates and which are considered valid biomarkers of dietary fatty acid intake are: *n*-3 PUFAs, such as linoleic acid (C18:2*n*-6), eicosapentaenoic acid (EPA) (C20:5*n*-3) and docosahexaenoic acid (DHA) (C22:6*n*-3), *n*-6 PUFAs, such as α-linoleic acid (18:3*n*-3), *trans *FAs and odd-numbered and branched-chain FAs [7]. Monounsaturated FAs (MUFAs) and saturated FAs (SFAs) do not reflect dietary intake patterns, with the exception of odd-numbered SFAs [6].

It is possible that some PUFAs, especially those of the *n*-3 group, are able to modulate the immune responses to *H. pylori*. Many studies report the effects of ingested FAs on molecular and cellular aspects of immunity [8]. SFAs are able to induce the activation of TLR2 and TLR4, whereas unsaturated FAs, such as *n*-3, inhibit TLR-mediated signaling pathways and gene expression [9,10]. In addition, any diet-induced variation in fatty acid composition of fat depots may influence directly the membrane organization of immune cells and result in impaired functionality [11,12]. In particular, dietary *n*-3 PUFAs alter T cell membrane microdomain composition and may therefore influence signaling complexes and modulate T cell activation in vivo [13,14].

Prostaglandins (PGs) play an important role in maintaining the gastro duodenal mucosal integrity [15]. PGs stimulate mucosal bicarbonate secretion, accelerate cell proliferation, enhance mucus secretion and mucosal blood flow, increase mucosal sulfhydryl groups and promote both lysosomal stability and the formation of mucosal phospholipids [16-19]. Dietary linoleic acid (C18:2*n*-6) is converted to arachidonic acid (C20:4*n*-6) which is the main unsaturated 20-carbon fatty acid used for the production of PGs via the cyclooxygenase pathway. Thus, an increased intake of linoleic acid might lead to an enhanced production of endogenous PGs.

The phospholipids of the gastric mucosa provide a hydrophobic lining that protects against extrinsic and intrinsic insults. The saturation level and the chain length of the FAs incorporated into these phospholipids influence both the membrane hydrophobicity and permeability [20].

Although some studies have examined the gastric mucosa in relation to *H. pylori*, little is known about the fatty acid composition of the gastric mucosa in relation to gastric ulceration. The aim of the present study is to compare the fatty acid composition of adipose tissue and gastric mucosa in subjects with gastric ulceration and subjects without evidence of ulceration.

## Methods

### Subjects

The study was conducted between June 2000 and November 2004 at the Gastroenterology Outpatient clinic of the University Hospital of Crete. Eligible patients were those with present or past abdominal complaints, who underwent upper gastrointestinal endoscopy screening. Patients were excluded if they were known to have concurrent illness, were on any medication or had taken H2 receptor antagonists, proton pump inhibitors, bismuth, antibiotics, non-steroidal anti-inflammatory drugs, or corticosteroids within 2 months of the examination. Fifty-two patients, all residents of the island of Crete, participated in the study. Patient ages ranged from 21 to 89 years (mean age 60, SD 16.9, median 64 years). The percentage of female subjects was similar in patients with and without gastric ulceration, being 9 out of 16 (56%) and 21 out of 36 (58%) respectively. All patients gave informed consent before the endoscopy, the endoscopic biopsy and the collection of adipose tissue samples. The study was approved by the Ethics Committee of the Medical School of the University of Crete.

The collection of the abdominal and buttock subcutaneous tissue samples as well as that of the gastric tissue was performed at the Department of Gastroenterology of the University Hospital of Heraklion, Crete.

All patients provided buttock subcutaneous tissue samples and 42 provided abdominal subcutaneous tissue samples. Gastric mucosa tissue samples were taken in a subset of 30 patients.

### Detection of gastric ulcer

The presence of gastric ulcer was determined by upper gastrointestinal tract endoscopy conducted in the Department of Gastroenterology of the University Hospital of Heraklion, Crete.

### Detection of *H. pylori *infection

The rapid urease CLO test was used for detecting *H. pylori *from gastric antral biopsy specimens. This test has been reported to have high sensitivity and specificity [21]. Biopsy specimens were taken from twenty-five patients, for histological examination. The histological results matched the results from the CLO test in all cases.

### Adipose tissue measures

Both abdominal and buttock subcutaneous adipose tissue samples were collected since there are reported differences in fatty acid content among abdominal and buttock depots [22]. Abdominal and buttock tissue samples were collected by aspiration, using the method described by Beynen and Katan [23]. The particular method is known to be rapid and safe, causing no more discomfort than a routine venipuncture [23]. Abdominal and buttock adipose tissue samples can be safely stored for up to 1.5 year without changes in the FA component [23]. Abdominal adipose tissue samples were taken from the left upper quadrant of the abdominal area, and in close proximity to the umbilicus. Buttock adipose tissue samples were taken from the left upper outer quadrant of the gluteal area. Both abdominal and buttock adipose tissue samples were taken through the use of 10 ml vacutainer tubes. Prior to aspiration, aspiration sites were sprayed with local anaesthetic (ethyl chloride).

Adipose tissue samples were stored at -80°C. Prior to analysis, the samples were thawed and the fat was transferred to 10 ml screw-capped tubes using Pasteur pipettes and several drops (~0.5 ml) of chloroform: methanol (2:1, v/v). Methyl esters of the fat component FAs (FAME) were prepared in the screw-capped vials according to the method described by Metcalfe et al (1966) [24]. Briefly, 20–30 mg of fat sample were saponified with 1.0 ml NaOH in methanol and the FAME were prepared with 14% boron trifluoride in methanol following extraction with hexane after washing with saturated NaCl. The hexane (upper layer) containing the FAME was transferred to GC vials and stored at -20°C until analysis. The FAME were separated on a 100 × 0.25 mm Id. SP-2560 fussed silica capillary column, coated with 0.25 μm of cyanopropyl silicone provided by SUPELCO (Bellefonte, PA, USA – SGE Australia), using a Shimadzu (Shimadzu Corporation Kyoto Japan) GC-17A/FID gas chromatograph equipped with an AOC-20I auto injector. The Class-VP chemstation software was used for identification and quantification of the peaks.

Baseline separation of over 50 FAME peaks was accomplished by means of mixed FAME standards (Sigma). The analytical conditions employed were as follows: 1 μl volume injected, helium carrier gas (1.1 ml/min), an injector temperature of 250°C, FID 260°C, split ratio 1:4 to 1:20 (depending on the sample quantity), and oven temperature from 140°C to 245°C with a stepped temperature program, within a total run time of 54 min.

The FAs extracted from adipose tissue lipids were C12:0, C14:0, C14:1*n*-5t, C14:1*n*-7t, C14:1*n*-9t, C15:0, C16:0, C16:1*n*-9t, C16:1*n*-7c, C16:1*n*-9c, C17:0, C18:0, sum of all *trans *C18:1, C18:1*n*-9c, C18:1*n*-11*cis*, C18:1*n*-12c, C18:1*n*-13c, C18:1*n*-14c, C18:2*n*-9t,12t, C18:2*n*-9c,12t, C18:2-9c,12c, C20:0, C18:3*n*-6, C20:1, C18:3*n*-3, C18:2 conjugated, C20:2*n*-9, C20:2*n*-6, C20:3*n*-6, C20:4*n*-6, C20:3*n*-3, C20:5*n*-3, C22:3*n*-3, C22:4, C22:5*n*-3 and C22:6*n*-3. All identified peaks were included in the statistical analyses.

In addition, the four fatty acid clusters SFA, MUFA, PUFA and *trans *FAs, the ratio of *n*-6:*n*-3 FAs, the ratios SFA:MUFA and SFA:PUFA and the sum of EPA and DHA were considered.

### Gastric mucosal tissue measures

Endoscopic pinch biopsy was used to obtain eight tissue samples from the pyloric antrum of each patient. Where a gastric ulcer was evident, the samples were collected from sites that were at a distance from the lesion.

Gastric mucosal tissue was stored at -80°C. Prior to analysis, the samples were thawed and the pinch biopsy was transferred to 10 ml screw-capped tubes using Pasteur pipettes and several drops (~0.5 ml) of chloroform: methanol (2:1, v/v). Methyl esters of the fat component FAs were prepared in the screw-capped vials according to the method described by Metcalfe et al (1966) [24]. Briefly, 20–30 mg of gastric tissue sample were saponified with 1.0 ml NaOH in methanol and the FAME were prepared with 2 mL 14% boron trifluoride in methanol following extraction with hexane after washing with 3.0 ml of saturated NaCl. The hexane (upper layer) containing the FAME was transferred to GC vials and stored at -20°C until analysis. The FAME of the gastric tissue were released from both triacyglycerols and phospholipids since the total quantity of the gastric tissue collected from each patient was relatively small. The FAME were separated on a 100 × 0.25 mm Id. SP-2560 fussed silica capillary column, coated with a 0.25 μm of cyanopropyl silicone provided by SUPELCO, using a Shimadzu GC-17A gas chromatograph equipped with an AOC-20I auto sampler and a FID. The Class-VP chemstation software was used for identification and quantification of the peaks.

The FAME extracted from some of the samples were also separated on a second capillary column in order to compare the results and decide on the most appropriate column to use. The column used was the BPX70 50 × 0.22 mm 0.25 U fusse silica column, coated with 0.2 μm of biscyanoproplyl polysiloxane, using a Shimadzu (Shimadzu Corporation Kyoto Japan) GC 2010GC gas chromatograph equipped with an AOC-20I auto sampler and an FID. When using the second column, the GC Solution software was used for peak identification and quantification. As the first column (SP-2560) provided a clearer analysis of the *cis*- and *trans*- isomers of C18:1 and C18:2, it was used for all analyses.

Baseline separation of the FAME peaks was accomplished by means of mixed FAME standards (Sigma). The analytical conditions employed were as follows: 1 μl volume injected, helium carrier gas used (20 cm/sec) (hydrogen for BPX70), injector temperature 250°C, FID temperature 250°C, split ratio 1:20 to 1:50 (depending on the sample quantity), and oven temperature from 140°C to 240°C (from 90°C to 230°C for BPX70) with a stepped temperature program within a total run time 60 min.

The FAs extracted from the gastric mucosa lipids were C12:0, C13:0, C14:0, C15:0, C16:0, C17:0, C18:0, C20:0, C22:0, C14:1*n*-9c, C16:1*n*-9c, C18:1*n*-9c, C18:1*n*-11c, C18:1*n*-12c, C18:1*n*-13c, C18:1*n*-14c, C20:1, C16:1*n*-5, C16:2*n*-4, C16:4*n*-1, C16:3*n*-4, C18:2-9c,12c, C18:3*n*-3, C18:3*n*-6, C18:2 conjugated, C20:3*n*-3, C20:3*n*-6, C20:4*n*-3, C20:4*n*-6, C20:5*n*-3, C21:5*n*-3, C22:4*n*-3, C22:5*n*-3, C22:6*n*-3, C22:5*n*-6, C16:1*n*-9t, C14:1*n*-5t, C14:1*n*-7t, sum of all *trans *C18:1, C18:2*n*-9t,12t, C18:2*n*-9c,12t and C18:2*n*-9t,12c. All identified peaks were included in the statistical analyses.

In addition, as in the adipose tissue, the four fatty acid clusters SFA, MUFA, PUFA and *trans *FAs, the ratio of *n*-6:*n*-3 FAs, the ratios SFA:MUFA and SFA:PUFA and the sum of EPA and DHA were considered.

### Data analysis

The chi-squared test of independence was applied to test for possible associations between categorical variables. Pearson's correlation coefficient was calculated to assess possible linear correlations between FAs from each of the three sites. Average levels of FAs were compared between the two gastric ulceration status groups using Student's t-test for independent samples at each of the three sites. In addition, analysis of covariance was undertaken to adjust for the possible confounding effect of age. Results were considered significant at the 5% level. The statistical package SPSS 15.0 was used throughout.

## Results

Of the 52 outpatients who participated in the study, 16 (31%) were found to have gastric ulceration. Twenty patients (39%) were CLO positive. CLO positivity was found to be strongly associated to gastric ulceration status (p = 0.003): eleven out of 16 patients with gastric ulceration were CLO positive (69%), whereas the number of CLO positive patients without gastric ulceration was nine out of 36 (25%). Age distribution also differed according to ulceration status: patients suffering from gastric ulceration had an average age of 67 years (SD = 15.2) whereas the mean age of patients without gastric ulceration was 58 years (SD = 17.4).

A positive linear correlation was found between the fatty acid clusters at each of the two adipose tissue sites: SFA (r = 0.73, p < 0.0001), MUFA (r = 0.81, p < 0.0001), PUFA (r = 0.84, p < 0.0001), *n*-3 cluster (r = 0.54, p < 0.0001), *n*-6 cluster (r = 0.83, p < 0.0001), *trans *(r = 0.612, p < 0.0001), MUFA:PUFA ratio (r = 0.86, p < 0.0001), MUFA:SFA ratio (r = 0.74, p < 0.0001), *n*-3:*n*-6 ratio (r = 0.63, p < 0.0001) and the sum of EPA and DHA (r = 0.88, p < 0.0001) using measurements from the 42 subjects who had samples from both sites.

Weak evidence of a positive correlation was found between adipose tissue and gastric mucosa for the *n*-6 cluster (buttock r = 0.37, p = 0.043, n = 30) and the *trans *cluster (abdomen r = 0.41, p = 0.047, n = 23). No other statistically significant correlations were detected between adipose and gastric tissue.

Average fatty acid composition was not found to differ between subjects with and without gastric ulceration in the two adipose tissue sites. Differences in average fatty acid composition in the gastric mucosa were, however, detected. Details are provided in Table 1. In summary, statistically significant differences were found for the SFAs C13:0 and C15:0 with higher average levels in patients with gastric ulceration. Mean levels of the following MUFAs were also found to differ to a statistically significant extent between the two groups: C14:1*n*-9c, C16:1*n*-5, C16:4*n*-1, C18:1*n*-12c and C20:1 with higher average levels of C14:1*n*-9c and C20:1 and lower levels of C16:1*n*-5, C16:4*n*-1 and C18:1*n*-12c in patients with gastric ulceration. The PUFAs that differed were the following: C16:2*n*-4, C16:3*n*-4, C20:3*n*-3, C20:4*n*-6, C21:5*n*-3, C22:4*n*-3 and the *trans *C18:2*n*-9c,12t with higher average levels of C16:2*n*-4 in ulcer positive patients and higher average levels of C16:3*n*-4, C20:3*n*-3, C20:4*n*-6, C21:5*n*-3, C22:4*n*-3 and C18:2*n*-9c,12t in ulcer negative patients. Details are provided in Table 1.

**Table 1:** Average levels of fatty acids extracted from the gastric mucosa lipids according to the presence (N = 11) or absence (N = 19) of peptic ulceration.

Fatty acid	Ulcer present (N = 11)	No ulcer present (N = 19)	**95% Confidence interval**.	p-value	p-value adjusted for age
	Mean (SE)	Mean (SE)			
SFA^a^	30.12 (0.540)	28.31 (0.668)	-0.179 to 3.806	0.073	0.069
MUFA^b^	27.17 (0.735)	25.87 (0.491)	-0.442 to 3.052	0.137	0.156
PUFA^c^	39.19 (0.825)	41.01 (0.510)	-3.701 to 0.052	0.056	0.090
*n*-3*	17.72 (1.407)	18.88 (0.550)	-3.787 to 1.468	0.374	0.410
*n*-6**	18.01 (0.768)	18.07 (0.503)	-1.869 to 1.740	0.942	0.856
*n*-6/*n*-3	1.09 (0.100)	0.98 (0.045)	-0.080 to 0.313	0.237	0.205
MUFA/SFA	0.91 (0.034)	0.92 (0.020)	-0.087 to 0.061	0.729	0.675
MUFA/PUFA	0.70 (0.028)	0.63 (0.019)	0.004 to 0.131	0.065	0.089
EPA† + DHA‡	14.34 (1.306)	15.14 (0.550)	-3.305 to 1.698	0.516	0.560
Total trans	0.94 (0.074)	0.98 (0.054)	-0.220 to 0.150	0.702	0.700
					
Saturated fatty acids					
C12:0	0.36 (0.046)	0.31 (0.039)	-0.087 to 0.169	0.519	0.837
C13:0	0.39 (0.077)	0.17 (0.031)	0.081 to 0.371	**0.003**	**0.005**
C14:0	0.65 (0.071)	0.53 (0.040)	-0.029 to 0.279	0.108	0.088
C15:0	1.92 (0.053)	1.56 (0.071)	0.158 to 0.576	**0.001**	**0.002**
					
C16:0	17.38 (0.344)	16.40 (0.390)	-0.207 to 2.155	0.103	0.072
C17:0	0.36 (0.040)	0.31 (0.032)	-0.058 to 0.152	0.371	0.481
C18:0	8.03 (0.292)	8.28 (0.364)	-1.333 to 0.834	0.641	0.672
C20:0	0.23 (0.051)	0.16 (0.012)	-0.020 to 0.148	0.134	0.190
C22:0	0.51 (0.131)	0.28 (0.055)	-0.018 to 0.481	0.069	0.070
					
Monounsaturated fatty acids					
C14:1*n*-9c	0.25 (0.028)	0.17 (0.007)	0.035 to 0.130	**0.001**	**0.001**
C16:1*n*-5	0.09 (0.019)	0.17 (0.019)	-0.133 to -0.013	**0.018**	**0.010**
C16:1*n*-9c	0.55 (0.057)	0.55 (0.044)	-0.153 to 0.142	0.943	0.867
C16 :4*n*-1	0.60 (0.056)	0.78 (0.046)	-0.332 to -0.027	**0.022**	**0.020**
					
C18:1*n*-9c	22.38 (0.608)	21.19 (0.485)	-0.428 to 2.799	0.144	0.169
C18:1*n*-11c	2.09 (0.042)	2.00 (0.057)	-0.073 to 0.263	0.259	0.273
C18:1*n*-12c	0.09 (0.009)	0.13 (0.009)	-0.066 to -0.011	**0.006**	**0.012**
C18:1*n*-13c	0.04 (0.006)	0.02 (0.006)	-0.001 to 0.035	0.059	**0.035**
C18:1*n*-14c	0.14 (0.018)	0.13 (0.009)	-0.032 to 0.041	0.814	0.615
C20:1	0.20 (0.030)	0.11 (0.009)	0.037 to 0.140	**0.001**	**0.001**
					
Polyunsaturated fatty acids					
C16:2*n*-4	0.55 (0.057)	0.33 (0.031)	0.973 to 0.339	**0.001**	**0.001**
C16:3*n*-4	1.13 (0.079)	1.45 (0.089)	-0.592 to -0.053	**0.021**	**0.014**
C18:2-9c,12c	16.97 (0.864)	16.57 (0.514)	-1.521 to 2.328	0.671	0.497
C18:3*n*-3	0.34 (0.056)	0.47 (0.067)	-0.331 to 0.072	0.200	0.167
C18:3*n*-6	0.14 (0.018)	0.15 (0.023)	-0.077 to 0.058	0.778	0.700
C18:2 conjugated	0.10 (0.008)	0.08 (0.005)	-0.002 to 0.032	0.095	0.061
C20:3*n*-3	0.04 (0.01)	0.34 (0.07)	-0.493 to -0.092	**0.006**	**0.004**
C20:3*n*-6	0.05 (0.008)	0.04 (0.010)	-0.014 to 0.044	0.307	0.241
C20:4*n*-3	2.20 (0.114)	1.91 (0.118)	-0.076 to 0.655	0.116	0.110
C20:4*n*-6	0.11 (0.018)	0.38 (0.084)	-0.492 to -0.035	**0.025**	**0.018**
C20:5*n*-3	11.58 (1.119)	12.53 (0.440)	-3.043 to 1.147	0.362	0.433
C21:5*n*-3	0.13 (0.014)	0.23 (0.015)	-0.148 to -0.057	**<0.0001**	**<0.0001**
C22:4*n*-3	0.15 (0.030)	0.22 (0.017)	-0.134 to -0.001	**0.045**	**0.047**
C22:5*n*-3	0.67 (0.136)	0.79 (0.036)	-0.350 to 0.109	0.293	0.373
C22:6*n*-3	2.75 (0.222)	2.61 (0.223)	-0.549 to 0.838	0.673	0.792
C22:5*n*-6	0.73 (0.145)	0.94 (0.053)	-0.474 to 0.055	0.117	0.139
					
trans					
C14:1*n*-5t	0.09 (0.017)	0.11 (0.017)	-0.080 to 0.027	0.318	0.199
C14:1*n*-7t	0.33 (0.046)	0.28 (0.032)	-0.060 to 0.163	0.351	0.514
C16:1*n*-9t	0.18 (0.027)	0.21 (0.023)	-0.107 to 0.041	0.372	0.374
C18:1*n*-t ^1^	0.23 (0.012)	0.32 (0.038)	-0.190 to 0.019	0.108	0.162
C18:2*n*-9t,12t	0.12 (0.029)	0.06 (0.021)	-0.014 to 0.130	0.111	**0.047**
C18:2*n*-9c,12t	0.17 (0.039)	0.42 (0.068)	-0.437 to -0.050	**0.015**	**0.009**
C18:2*n*-9t,12c	0.12 (0.009)	0.27 (0.068)	-0.333 to 0.037	0.114	0.063

^a ^SFA = sum of saturated fatty acids

^b ^MUFA = sum of monounsaturated fatty acids

^c ^PUFA = sum of polyunsaturated fatty acids

* sum of *n*-3 fatty acids

** sum of *n*-6 fatty acids

†Eicosapentaenoic acid C20:5*n*-3 (EPA)

‡Docosahexaenoic acid C22:6*n*-3 (DHA)

^1 ^All trans C18:1 (C18:1n-6t, C18:1n-7t, C18:1n-8t and C18:1n-9t)

In subjects who were not found to have a gastric ulcer, abdominal tissue fatty acid composition was found to differ according to CLO status, there being higher levels of the SFAs C12:0 and C20:0 in abdominal tissue in CLO positive patients. In contrast, the average levels of C20:4*n*-6, C20:3*n*-3, C20:5*n*-3, C22:4, C22:5*n*-3 and C22:6*n*-3 were found to be significantly lower in CLO positive patients. Details are provided in Table 2.

**Table 2:** Average levels of fatty acids extracted from adipose tissue lipids in patients without gastric ulcer according to CLO status. (N = 30)^1^

Abdominal tissue				
				
Fatty acid	CLO + (N = 7)	CLO - (N = 23)	**95% Confidence interval**.	p-value
	Mean (SE)	Mean (SE)		
*n*-3*	1.02 (0.065)	1.27 (0.054)	-0.469 to -0.038	**0.023**
C12:0	0.34 (0.091)	0.19 (0.020)	0.026 to 0.274	**0.019**
C20:0	0.16 (0.016)	0.12 (0.009)	0.001 to 0.075	**0.044**
C20:4*n*-6	0.32 (0.040)	0.44 (0.026)	-0.224 to -0.009	**0.035**
C20:3*n*-3	0.03 (0.005)	0.05 (0.004)	-0.035 to -0.001	**0.037**
C20:5*n*-3	0.02 (0.002)	0.04 (0.003)	-0.026 to -0.006	**0.002**
C22:4	0.03 (0.002)	0.04 (0.003)	-0.023 to -0.003	**0.012**
C22:5*n*-3	0.11 (0.011)	0.18 (0.011)	-0.107 to -0.023	**0.004**
C22:6n-3	0.10 (0.008)	0.19 (0.015)	-0.142 to -0.031	**0.003**

**Buttock tissue**				
				
**Fatty acid**	**CLO + (N = 7)**	**CLO - (N = 23)**	**95% Confidence interval**.	**p-value**
	**Mean (SE)**	**Mean (SE)**		

C12:0	0.26 (0.061)	0.16 (0.017)	0.004 to 0.186	**0.042**
C20:0	0.13 (0.014)	0.09 (0.005)	0.017 to 0.065	**0.001**
C16:1*n*-7c	0.79 (0.048)	0.93 (0.034)	-0.268 to -0.003	**0.046**
C20:1	0.44 (0.034)	0.38 (0.013)	0.003 to 0.123	**0.039**
C20:3*n*-6	0.20 (0.029)	0.27 (0.016)	-0.138 to -0.007	**0.030**
C20:4*n*-6	0.34 (0.051)	0.45 (0.026)	-0.217 to 0.001	**0.050**
C22:5*n*-3	0.13 (0.012)	0.18 (0.011)	-0.090 to -0.011	**0.015**
C22:6*n*-3	0.12 (0.013)	0.18 (0.012)	-0.112 to -0.021	**0.006**

* sum of *n*-3 fatty acids

^1 ^Only comparisons that are statistically significant at the 5% level are presented in the above Table (p < 0.05)

Regarding the fatty acid composition of the buttock tissue, there seems to be a possible relationship with the CLO status in subjects without gastric ulcer. Levels of the SFAs C20:0 and C12:0 and the MUFA C20:1 were higher in CLO positive patients, whereas the levels of C16:1*n*-7c, C20:3*n*-6, C20:4*n*-6, C22:5*n*-3 and C22:6*n*-3 were lower. Details are provided in Table 2.

## Discussion

In the present study, no notable differences in adipose fatty acid composition were found between subjects with and without gastric ulceration. These findings are in agreement with those of a study that examined the possible preventative role of linoleic (C18:2*n*-6) fatty acid in gastric ulceration. The authors came to the conclusion that there was no significant difference in the adipose tissue content of linoleic acid between patients with gastric ulcer disease and matched control subjects [25]. Our findings are, however, in contrast to the results of Grant et al (1990) and Prichard et al (1988) who presented a possible preventing role of dietary intake of linoleic fat acid and olive oil in gastric ulceration [26,27].

In our study, ulcer-negative subjects were found to differ in adipose tissue fatty acid composition according to CLO status, there being higher levels of C20:4*n*-6, C20:3*n*-3, C20:5*n*-3 (EPA), C22:4, C22:5*n*-3 and C22:6*n*-3 (DHA) in abdominal samples and C16:1*n*-7*cis*, C20:3*n*-6, C20:4*n*-6, C22:5*n*-3 and C22:6*n*-3 in buttock samples in CLO negative patients.

It is possible that the consumption of PUFAs inhibits the growth of *H. pylori *and the colonization of the gastric mucosa, a hypothesis that is in agreement with previous studies showing that ingested dietary PUFAs inhibit the growth of *H. pylori *in vitro [5,28]. These studies suggest that the inhibitory effect of PUFAs to *H. pylori *is due: (a) to the disruption of the bacterial outer lipid membrane which leads to cell lysis and (b) to the polyunsaturated induced increased permeability of the bacterial cell membranes which leads to dissipated concentration gradients between the organism and its environment, such as those for hydrogen ions with fatal outcomes for *H. pylori *[5].

It is also possible that the higher levels of some PUFAs, especially those of the *n*-3 group, in CLO negative patients reflect the role of FAs to the modulation of immune responses to *H. pylori*. Adipose tissue functions are, in fact, crucial in protective immunity against noxious agents such as *H pylori*. Mueller et al (2003) have concluded that a number of genes specifically expressed in fat cells/adipocytes, tightly correlate with protection from *H. pylori*. Among these are genes for three adipocyte-specific cytokines, the so-called "adipokines," adipsin, resistin, and adiponectin [8]. It has also been suggested that the secreted adipokines are likely mediators of crosstalk with lymphocytes. This paracrine relationship between adipose and lymphoid tissues possibly explains the link between the production and secretion of adipokines and a protective response against *H. pylori *[8]. It is possible that secreted adipocyte factors stimulate the effector functions of resident T cell populations [8]. This hypothesis is supported by numerous studies demonstrating an essential role of CD4^+ ^T cells in the protection against *H. pylori *[29-31].

Findings from dietary intervention studies lead, however, to conclusion that PUFAs, when orally ingested as dietary supplements, do not inhibit either the colonization of the mucosa by *H. pylori *or the inflammatory changes characteristic of *H. pylori *gastritis [28]. It may be that the absorption and distribution of PUFAs derived from capsules differ from those naturally present in the diet. Further research could investigate whether the long term supply of digested PUFA foods, as estimated by the adipose tissue fatty acid composition, is of greater effectiveness against *H. pylori *infection than the short term per os supply of high doses of PUFA.

To the best of our knowledge, this is the first literature report of a possible association between fatty acid composition of the gastric mucosa and the presence of gastric ulceration. It was found that the MUFAs C18:1*n*-12c, C16:1*n*-5 and C16:4*n*-1 and the PUFAs C16:3*n*-4, C20:3*n*-3, C20:4*n*-6, C21:5*n*-3, C22:4*n*-3 and C18:2*n*-9c,12t were present at somewhat higher levels in the gastric mucosa of ulcer-negative patients, leading to the hypothesis that some PUFAs may possibly play a possible preventive role in gastric ulceration. Additionally, there were significantly higher proportions of C13:0, C15:0, C14:1*n*-9c and C20:1 in the gastric mucosa of patients with gastric ulcer disease, also indicating a possible protective role for some FAs versus others in gastric ulceration.

Arachidonic acid (C20:4*n*-6) is incorporated in membrane phospholipids. Surface active phospholipids, especially phosphatidylcholine, play an important role in the first line of defense of the stomach, most likely because of their hydrophobic properties. Both membrane hydrophobicity and permeability are known to be influenced by the saturation level and chain length of FAs that composes the surface active phospholipids [20].

In addition, arachidonic acid is a precursor fatty acid in the prostaglandin synthesis pathway. PGs of the E, A and I series have been shown to suppress basal and stimulated gastric acid secretion in rats and normal human subjects [32]. Furthermore, it has been demonstrated that PGs of the E type are capable of preventive formation of gastric ulcers in rats induced by a wide variety of experimental techniques [33]. Tarnawski et al (1987) also showed a significant cytoprotective effect of oral administration of linoleic acid on ethanol-induced gastric injury, an effect significantly reduced by pre-treatment with oral indomethacin, a known inhibitor of prostaglandin synthetase [34].

Both the design of our study and the results themselves, however, cannot lead to the conclusion of a definitive relation between fatty acid composition and gastric ulceration status. In addition, it is known that when multiple comparisons are undertaken, there is an effective increase in the significance level. Adjusting for multiple comparisons using the Bonferroni correction (adjusted alpha = 0.001), results in only C14:1*n*-9 c, C20:1, C16:2*n*-4 and C21:5*n*-3 remaining statistically significant at the unadjusted 5% level (Table 1). None of these fatty acids contributed more than 1% on average to the total fatty acid content. The biological relevance of the statistically significant results is not clear. The role that CLO status may play in the relation between fatty acid composition and the presence of gastric ulceration could not be investigated (or accounted for in the statistical analysis) as 18 of the 19 subjects without a gastric ulcer from whom a gastric mucosal sample was taken, were CLO negative. Since it is known that *H. pylori *infection alters not only the gastric mucosal phosphatidylcholine content, but also its fatty acid composition, which may consequently cause the gastric mucosal barrier to weaken, further investigation is needed in order to clarify the relation of the fatty acid composition of the gastric mucosa to *H. pylori *colonization.

## Conclusion

Certain MUFAs and PUFAs of the gastric mucosa appear to be present in somewhat higher proportions in ulcer negative patients. These findings seem to suggest a possible protective role of the gastric mucosa FAs in gastric ulceration, although statistically significant differences were found only in certain minor FAs i.e. FAs contributing only a very small fraction of the total fatty acid contents. The higher average levels of EPA and DHA in abdominal and buttock adipose tissue in CLO negative, ulcer negative patients, may also suggest that dietary FAs could inhibit *H. pylori *growth. Further studies are required to ascertain whether such FAs do, in fact, play a role in the prevention of gastric ulceration.

## Abbreviations

*H. pylori*: *Helicobacter pylori*; CLO: Campylobacter-Like Organism test (rapid urease test for *H. pylori *infection); EPA: eicosapentaenoic acid; DHA: docosahexaenoic acid; HLA: human leukocyte antigen; FAs: fatty acids; PGs: prostaglandins; PUFA: polyunsaturated fatty acids; MUFA: monounsaturated fatty acids; SFA: saturated fatty acids; GS: gas chromatography; FAME: fatty acid methyl esters; FID: Flame ionization detector; TLR: Toll-like receptors; APC: Antigen-presenting cell.

## Competing interests

The authors declare that they have no competing interests.

## Authors' contributions

VAP collected the adipose tissue samples, participated in the design and coordination of the study and wrote the manuscript. JM performed the statistical analysis. MK carried out the capillary gas chromatography. MR performed the upper gastrointestinal tract endoscopies and collected the gastric mucosa samples. AK and EK conceived of the study and participated in its design and coordination. All authors read and approved the final manuscript.

## Pre-publication history

The pre-publication history for this paper can be accessed here:

http://www.biomedcentral.com/1471-230X/9/9/prepub
